# Supplementary material for: Studies on the formation of formaldehyde during 2-ethylhexyl 4-(dimethylamino)benzoate demethylation in the presence of reactive oxygen and chlorine species
Source: Environ Sci Pollut Res Int. 2017 Jan 29;24(9):8049–61. doi: 10.1007/s11356-017-8477-8 (PMC5384958; doi:10.1007/s11356-017-8477-8)
Supplement: Supplementary file 1 — (PDF 641 kb) [file 11356_2017_8477_MOESM1_ESM.pdf]

**Online Resource**

# Studies on the formation of formaldehyde during 2-ethylhexyl 4-(dimethylamino)benzoate demethylation in the presence of reactive oxygen and chlorine species

Waldemar Studziński[1], Alicja Gackowska[1], Maciej Przybyłek[2] and Jerzy Gaca[1]
[1]Faculty of Chemical Technology and Engineering, University of Technology and Life Science, Seminaryjna 3, 85-326 Bydgoszcz, Poland;
[2]Department of Physical Chemistry, Faculty of Pharmacy, Ludwik Rydygier Collegium Medicum in Bydgoszcz, Nicolaus Copernicus University in Toruń, Kurpińskiego 5, 85-950 Bydgoszcz, Poland

## I. Selected quantum-chemical calculations results

**Table S1** Bond lengths of optimized 2-ethylhexyl 4-(dimethylamino)benzoate (ODPABA) molecule (atoms numbering according to Fig. 3a)

| No. | Atom1 | Atom2 | Cyclicity | Length [Å] |
|---|---|---|---|---|
| 1 | H1 | C36 | acyclic | 1.0905 |
| 2 | H2 | C36 | acyclic | 1.1005 |
| 3 | H3 | C36 | acyclic | 1.0964 |
| 4 | H4 | C47 | acyclic | 1.0906 |
| 5 | H5 | C47 | acyclic | 1.1004 |
| 6 | H6 | C47 | acyclic | 1.0963 |
| 7 | H7 | C34 | acyclic | 1.0827 |
| 8 | H8 | C33 | acyclic | 1.0852 |
| 9 | H9 | C30 | acyclic | 1.0828 |
| 10 | H10 | C31 | acyclic | 1.0843 |
| 11 | H11 | C43 | acyclic | 1.0949 |
| 12 | H12 | C43 | acyclic | 1.0963 |
| 13 | H13 | C39 | acyclic | 1.1003 |
| 14 | H14 | C41 | acyclic | 1.0957 |
| 15 | H15 | C41 | acyclic | 1.1000 |
| 16 | H16 | C45 | acyclic | 1.0967 |
| 17 | H17 | C45 | acyclic | 1.0954 |
| 18 | H18 | C45 | acyclic | 1.0953 |
| 19 | H19 | C42 | acyclic | 1.0998 |
| 20 | H20 | C40 | acyclic | 1.0993 |
| 21 | H21 | C40 | acyclic | 1.0988 |
| 22 | H22 | C42 | acyclic | 1.0983 |
| 23 | H23 | C46 | acyclic | 1.0965 |
| 24 | H24 | C44 | acyclic | 1.0990 |
| 25 | H25 | C44 | acyclic | 1.0990 |
| 26 | H26 | C46 | acyclic | 1.0954 |
| 27 | H27 | C46 | acyclic | 1.0966 |
| 28 | C28 | C32 | acyclic | 1.4795 |
| 29 | C28 | O37 | acyclic | 1.3580 |
| 30 | C28 | O38 | acyclic | 1.2212 |
| 31 | C29 | C34 | cyclic | 1.4194 |

| No. | Atom1 | Atom2 | Cyclicity | Length [Å] |
|---|---|---|---|---|
| **32** | C29 | C30 | cyclic | 1.4188 |
| **33** | C29 | N35 | acyclic | 1.3810 |
| **34** | C30 | C31 | cyclic | 1.3888 |
| **35** | C31 | C32 | cyclic | 1.4039 |
| **36** | C32 | C33 | cyclic | 1.4038 |
| **37** | C33 | C34 | cyclic | 1.3872 |
| **38** | N35 | C47 | acyclic | 1.4554 |
| **39** | N35 | C36 | acyclic | 1.4552 |
| **40** | O37 | C43 | acyclic | 1.4451 |
| **41** | C39 | C40 | acyclic | 1.5538 |
| **42** | C39 | C41 | acyclic | 1.5428 |
| **43** | C39 | C43 | acyclic | 1.5292 |
| **44** | C40 | C42 | acyclic | 1.5366 |
| **45** | C41 | C45 | acyclic | 1.5332 |
| **46** | C42 | C44 | acyclic | 1.5342 |
| **47** | C44 | C46 | acyclic | 1.5331 |

**Table S2** Bond angles of optimized 2-ethylhexyl 4-**(**dimethylamino)benzoate (ODPABA) molecule (atoms numbering according to Fig. 3a)

| No. | Atom1 | Atom2 | Atom3 | Angle [°] |
|---|---|---|---|---|
| **1** | C32 | C28 | O37 | 112.81 |
| **2** | C32 | C28 | O38 | 124.66 |
| **3** | O37 | C28 | O38 | 122.53 |
| **4** | C34 | C29 | C30 | 117.36 |
| **5** | C34 | C29 | N35 | 121.31 |
| **6** | C30 | C29 | N35 | 121.34 |
| **7** | H9 | C30 | C29 | 120.41 |
| **8** | H9 | C30 | C31 | 118.56 |
| **9** | C29 | C30 | C31 | 121.03 |
| **10** | H10 | C31 | C30 | 119.31 |
| **11** | H10 | C31 | C32 | 119.49 |
| **12** | C30 | C31 | C32 | 121.20 |
| **13** | C28 | C32 | C31 | 123.21 |
| **14** | C28 | C32 | C33 | 118.69 |
| **15** | C31 | C32 | C33 | 118.10 |
| **16** | H8 | C33 | C32 | 118.57 |
| **17** | H8 | C33 | C34 | 120.04 |
| **18** | C32 | C33 | C34 | 121.39 |
| **19** | H7 | C34 | C29 | 120.45 |
| **20** | H7 | C34 | C33 | 118.64 |
| **21** | C29 | C34 | C33 | 120.91 |
| **22** | C29 | N35 | C47 | 119.96 |
| **23** | C29 | N35 | C36 | 119.97 |
| **24** | C47 | N35 | C36 | 118.78 |

| No. | Atom1 | Atom2 | Atom3 | Angle [°] |
|---|---|---|---|---|
| **25** | H1 | C36 | H2 | 108.21 |
| **26** | H1 | C36 | H3 | 107.64 |
| **27** | H1 | C36 | N35 | 109.09 |
| **28** | H2 | C36 | H3 | 108.15 |
| **29** | H2 | C36 | N35 | 112.39 |
| **30** | H3 | C36 | N35 | 111.21 |
| **31** | C28 | O37 | C43 | 116.24 |
| **32** | H13 | C39 | C40 | 108.42 |
| **33** | H13 | C39 | C41 | 108.02 |
| **34** | H13 | C39 | C43 | 106.94 |
| **35** | C40 | C39 | C41 | 113.18 |
| **36** | C40 | C39 | C43 | 108.98 |
| **37** | C41 | C39 | C43 | 111.06 |
| **38** | H20 | C40 | H21 | 105.44 |
| **39** | H20 | C40 | C39 | 109.69 |
| **40** | H20 | C40 | C42 | 107.96 |
| **41** | H21 | C40 | C39 | 109.47 |
| **42** | H21 | C40 | C42 | 109.38 |
| **43** | C39 | C40 | C42 | 114.49 |
| **44** | H14 | C41 | H15 | 106.27 |
| **45** | H14 | C41 | C39 | 108.52 |
| **46** | H14 | C41 | C45 | 109.05 |
| **47** | H15 | C41 | C39 | 108.44 |
| **48** | H15 | C41 | C45 | 109.36 |
| **49** | C39 | C41 | C45 | 114.86 |
| **50** | H19 | C42 | H22 | 105.98 |
| **51** | H19 | C42 | C40 | 108.95 |
| **52** | H19 | C42 | C44 | 109.17 |
| **53** | H22 | C42 | C40 | 109.81 |
| **54** | H22 | C42 | C44 | 109.25 |
| **55** | C40 | C42 | C44 | 113.41 |
| **56** | H11 | C43 | H12 | 107.66 |
| **57** | H11 | C43 | O37 | 108.42 |
| **58** | H11 | C43 | C39 | 111.19 |
| **59** | H12 | C43 | O37 | 108.95 |
| **60** | H12 | C43 | C39 | 111.56 |
| **61** | O37 | C43 | C39 | 108.97 |
| **62** | H24 | C44 | H25 | 106.04 |
| **63** | H24 | C44 | C42 | 109.20 |
| **64** | H24 | C44 | C46 | 109.43 |
| **65** | H25 | C44 | C42 | 109.21 |
| **66** | H25 | C44 | C46 | 109.42 |
| **67** | C42 | C44 | C46 | 113.28 |
| **68** | H16 | C45 | H17 | 107.59 |

| No. | Atom1 | Atom2 | Atom3 | Angle [°] |
|---|---|---|---|---|
| **69** | H16 | C45 | H18 | 107.68 |
| **70** | H16 | C45 | C41 | 111.23 |
| **71** | H17 | C45 | H18 | 107.37 |
| **72** | H17 | C45 | C41 | 110.71 |
| **73** | H18 | C45 | C41 | 112.06 |
| **74** | H23 | C46 | H26 | 107.66 |
| **75** | H23 | C46 | H27 | 107.53 |
| **76** | H23 | C46 | C44 | 111.16 |
| **77** | H26 | C46 | H27 | 107.67 |
| **78** | H26 | C46 | C44 | 111.43 |
| **79** | H27 | C46 | C44 | 111.20 |
| **80** | H4 | C47 | H5 | 108.23 |
| **81** | H4 | C47 | H6 | 107.64 |
| **82** | H4 | C47 | N35 | 109.08 |
| **83** | H5 | C47 | H6 | 108.14 |
| **84** | H5 | C47 | N35 | 112.42 |
| **85** | H6 | C47 | N35 | 111.17 |

**Table S3** Dihedral angles of optimized 2-ethylhexyl 4-(dimethylamino)benzoate (ODPABA) molecule (atoms numbering according to Fig. 3a)

| No. | Atom1 | Atom2 | Atom3 | Atom4 | Dihedral angle [°] |
|---|---|---|---|---|---|
| 1 | O37 | C28 | C32 | C31 | -0.85 |
| 2 | O37 | C28 | C32 | C33 | 179.13 |
| 3 | O38 | C28 | C32 | C31 | 179.05 |
| 4 | O38 | C28 | C32 | C33 | -0.96 |
| 5 | C32 | C28 | O37 | C43 | 178.72 |
| 6 | O38 | C28 | O37 | C43 | -1.19 |
| 7 | C30 | C29 | C34 | H7 | -179.07 |
| 8 | C30 | C29 | C34 | C33 | 1.01 |
| 9 | N35 | C29 | C34 | H7 | 1.27 |
| 10 | N35 | C29 | C34 | C33 | -178.66 |
| 11 | C34 | C29 | C30 | H9 | 179.15 |
| 12 | C34 | C29 | C30 | C31 | -0.98 |
| 13 | N35 | C29 | C30 | H9 | -1.19 |
| 14 | N35 | C29 | C30 | C31 | 178.68 |
| 15 | C34 | C29 | N35 | C47 | -6.59 |
| 16 | C34 | C29 | N35 | C36 | -173.48 |
| 17 | C30 | C29 | N35 | C47 | 173.76 |
| 18 | C30 | C29 | N35 | C36 | 6.87 |
| 19 | H9 | C30 | C31 | H10 | 0.17 |
| 20 | H9 | C30 | C31 | C32 | -179.74 |
| 21 | C29 | C30 | C31 | H10 | -179.71 |
| 22 | C29 | C30 | C31 | C32 | 0.38 |
| 23 | H10 | C31 | C32 | C28 | 0.30 |

| | | | | | |
|---|---|---|---|---|---|
| 24 | H10 | C31 | C32 | C33 | -179.69 |
| 25 | C30 | C31 | C32 | C28 | -179.79 |
| 26 | C30 | C31 | C32 | C33 | 0.22 |
| 27 | C28 | C32 | C33 | H8 | -0.37 |
| 28 | C28 | C32 | C33 | C34 | 179.82 |
| 29 | C31 | C32 | C33 | H8 | 179.62 |
| 30 | C31 | C32 | C33 | C34 | -0.19 |
| 31 | H8 | C33 | C34 | H7 | -0.17 |
| 32 | H8 | C33 | C34 | C29 | 179.76 |
| 33 | C32 | C33 | C34 | H7 | 179.63 |
| 34 | C32 | C33 | C34 | C29 | -0.44 |
| 35 | C29 | N35 | C47 | H4 | 177.95 |
| 36 | C29 | N35 | C47 | H5 | -62.00 |
| 37 | C29 | N35 | C47 | H6 | 59.40 |
| 38 | C36 | N35 | C47 | H4 | -15.00 |
| 39 | C36 | N35 | C47 | H5 | 105.05 |
| 40 | C36 | N35 | C47 | H6 | -133.55 |
| 41 | C29 | N35 | C36 | H1 | -178.47 |
| 42 | C29 | N35 | C36 | H2 | 61.52 |
| 43 | C29 | N35 | C36 | H3 | -59.90 |
| 44 | C47 | N35 | C36 | H1 | 14.48 |
| 45 | C47 | N35 | C36 | H2 | -105.53 |
| 46 | C47 | N35 | C36 | H3 | 133.06 |
| 47 | C28 | O37 | C43 | H11 | -60.19 |
| 48 | C28 | O37 | C43 | H12 | 56.72 |
| 49 | C28 | O37 | C43 | C39 | 178.65 |
| 50 | H13 | C39 | C40 | H20 | 104.57 |
| 51 | H13 | C39 | C40 | H21 | -140.19 |
| 52 | H13 | C39 | C40 | C42 | -16.96 |
| 53 | C41 | C39 | C40 | H20 | -135.61 |
| 54 | C41 | C39 | C40 | H21 | -20.36 |
| 55 | C41 | C39 | C40 | C42 | 102.86 |
| 56 | C43 | C39 | C40 | H20 | -11.50 |
| 57 | C43 | C39 | C40 | H21 | 103.75 |
| 58 | C43 | C39 | C40 | C42 | -133.03 |
| 59 | H13 | C39 | C41 | H14 | -64.90 |
| 60 | H13 | C39 | C41 | H15 | -179.93 |
| 61 | H13 | C39 | C41 | C45 | 57.41 |
| 62 | C40 | C39 | C41 | H14 | 175.05 |
| 63 | C40 | C39 | C41 | H15 | 60.02 |
| 64 | C40 | C39 | C41 | C45 | -62.64 |
| 65 | C43 | C39 | C41 | H14 | 52.08 |
| 66 | C43 | C39 | C41 | H15 | -62.95 |
| 67 | C43 | C39 | C41 | C45 | 174.40 |
| 68 | H13 | C39 | C43 | H11 | -63.28 |

| | | | | | |
|---|---|---|---|---|---|
| 69 | H13 | C39 | C43 | H12 | 176.51 |
| 70 | H13 | C39 | C43 | O37 | 56.17 |
| 71 | C40 | C39 | C43 | H11 | 53.72 |
| 72 | C40 | C39 | C43 | H12 | -66.48 |
| 73 | C40 | C39 | C43 | O37 | 173.18 |
| 74 | C41 | C39 | C43 | H11 | 179.07 |
| 75 | C41 | C39 | C43 | H12 | 58.87 |
| 76 | C41 | C39 | C43 | O37 | -61.48 |
| 77 | H20 | C40 | C42 | H19 | -63.45 |
| 78 | H20 | C40 | C42 | H22 | -179.12 |
| 79 | H20 | C40 | C42 | C44 | 58.35 |
| 80 | H21 | C40 | C42 | H19 | -177.71 |
| 81 | H21 | C40 | C42 | H22 | 66.63 |
| 82 | H21 | C40 | C42 | C44 | -55.91 |
| 83 | C39 | C40 | C42 | H19 | 59.02 |
| 84 | C39 | C40 | C42 | H22 | -56.65 |
| 85 | C39 | C40 | C42 | C44 | -179.18 |
| 86 | H14 | C41 | C45 | H16 | 63.87 |
| 87 | H14 | C41 | C45 | H17 | -55.69 |
| 88 | H14 | C41 | C45 | H18 | -175.53 |
| 89 | H15 | C41 | C45 | H16 | 179.69 |
| 90 | H15 | C41 | C45 | H17 | 60.12 |
| 91 | H15 | C41 | C45 | H18 | -59.72 |
| 92 | C39 | C41 | C45 | H16 | -58.15 |
| 93 | C39 | C41 | C45 | H17 | -177.72 |
| 94 | C39 | C41 | C45 | H18 | 62.44 |
| 95 | H19 | C42 | C44 | H24 | 64.75 |
| 96 | H19 | C42 | C44 | H25 | -179.70 |
| 97 | H19 | C42 | C44 | C46 | -57.47 |
| 98 | H22 | C42 | C44 | H24 | -179.77 |
| 99 | H22 | C42 | C44 | H25 | -64.23 |
| 100 | H22 | C42 | C44 | C46 | 58.00 |
| 101 | C40 | C42 | C44 | H24 | -56.92 |
| 102 | C40 | C42 | C44 | H25 | 58.62 |
| 103 | C40 | C42 | C44 | C46 | -179.15 |
| 104 | H24 | C44 | C46 | H23 | -62.22 |
| 105 | H24 | C44 | C46 | H26 | 57.87 |
| 106 | H24 | C44 | C46 | H27 | 178.00 |
| 107 | H25 | C44 | C46 | H23 | -178.02 |
| 108 | H25 | C44 | C46 | H26 | -57.93 |
| 109 | H25 | C44 | C46 | H27 | 62.20 |
| 110 | C42 | C44 | C46 | H23 | 59.87 |
| 111 | C42 | C44 | C46 | H26 | 179.97 |
| 112 | C42 | C44 | C46 | H27 | -59.90 |

**Table S4** List of frequencies, reduced masses, force constants and IR intensities, calculated for optimized ODPABA molecule

| No | Frequencies [cm$^{-1}$] | Reduced mass [AMU] | Force constants [mDyne/Å] | IR intensity [km/mol] |
|---|---|---|---|---|
| **1** | 15.1631 | 3.8713 | 0.0005 | 0.6975 |
| **2** | 20.5029 | 3.2435 | 0.0008 | 1.0581 |
| **3** | 34.0201 | 2.7473 | 0.0019 | 4.5793 |
| **4** | 38.7171 | 3.5672 | 0.0032 | 1.3155 |
| **5** | 42.2815 | 2.5833 | 0.0027 | 1.0839 |
| **6** | 67.9098 | 4.2676 | 0.0116 | 2.1992 |
| **7** | 71.9738 | 1.7523 | 0.0053 | 0.1883 |
| **8** | 81.2458 | 2.4840 | 0.0097 | 0.1178 |
| **9** | 88.1116 | 3.1223 | 0.0143 | 1.2784 |
| **10** | 105.4334 | 2.5937 | 0.0170 | 0.9067 |
| **11** | 121.0605 | 2.5202 | 0.0218 | 0.3225 |
| **12** | 126.0891 | 2.3628 | 0.0221 | 0.1304 |
| **13** | 166.0810 | 2.8863 | 0.0469 | 0.2039 |
| **14** | 179.1672 | 2.2347 | 0.0423 | 0.4852 |
| **15** | 190.4113 | 1.9476 | 0.0416 | 1.3255 |
| **16** | 195.2767 | 1.9041 | 0.0428 | 1.0174 |
| **17** | 215.6932 | 2.3957 | 0.0657 | 8.2620 |
| **18** | 234.9577 | 1.8200 | 0.0592 | 4.7826 |
| **19** | 249.3847 | 1.4127 | 0.0518 | 2.0713 |
| **20** | 251.1058 | 1.4840 | 0.0551 | 0.7425 |
| **21** | 270.6109 | 1.7595 | 0.0759 | 0.6332 |
| **22** | 300.7940 | 5.3882 | 0.2872 | 10.7097 |
| **23** | 314.3886 | 3.9226 | 0.2284 | 1.0634 |
| **24** | 320.3445 | 3.8179 | 0.2308 | 2.8299 |
| **25** | 370.1971 | 2.3769 | 0.1919 | 0.4085 |
| **26** | 390.4327 | 2.7715 | 0.2489 | 0.6180 |
| **27** | 432.6829 | 3.0523 | 0.3367 | 0.0017 |
| **28** | 452.3379 | 2.8283 | 0.3410 | 0.5617 |
| **29** | 469.8858 | 3.7137 | 0.4831 | 5.4074 |
| **30** | 496.4195 | 3.1197 | 0.4530 | 7.0583 |
| **31** | 513.5783 | 2.5833 | 0.4015 | 20.0392 |
| **32** | 520.1591 | 3.1007 | 0.4943 | 17.4713 |
| **33** | 561.3670 | 4.3118 | 0.8006 | 3.6309 |
| **34** | 609.5726 | 4.4345 | 0.9708 | 13.9151 |
| **35** | 644.4602 | 7.1576 | 1.7515 | 4.1636 |
| **36** | 704.6661 | 3.2381 | 0.9473 | 32.3723 |
| **37** | 729.6401 | 1.0802 | 0.3388 | 7.0232 |
| **38** | 758.2935 | 5.1088 | 1.7308 | 5.4754 |
| **39** | 773.6519 | 5.3342 | 1.8811 | 71.2879 |
| **40** | 780.0289 | 1.1912 | 0.4270 | 4.4539 |
| **41** | 782.7223 | 1.4799 | 0.5342 | 6.7163 |

| No | Frequencies [cm$^{-1}$] | Reduced mass [AMU] | Force constants [mDyne/Å] | IR intensity [km/mol] |
|---|---|---|---|---|
| **42** | 808.5866 | 1.2607 | 0.4856 | 0.6037 |
| **43** | 825.1791 | 1.7710 | 0.7105 | 6.6252 |
| **44** | 835.9577 | 2.1484 | 0.8846 | 58.8164 |
| **45** | 864.5351 | 5.4850 | 2.4154 | 10.4753 |
| **46** | 899.9372 | 1.8128 | 0.8650 | 2.5986 |
| **47** | 928.0165 | 1.3052 | 0.6623 | 4.4040 |
| **48** | 956.4867 | 3.3574 | 1.8097 | 1.0167 |
| **49** | 962.6871 | 3.2190 | 1.7577 | 67.6974 |
| **50** | 968.2586 | 1.3818 | 0.7633 | 3.5246 |
| **51** | 972.2200 | 1.3300 | 0.7407 | 1.1339 |
| **52** | 980.3835 | 1.3403 | 0.7590 | 0.0449 |
| **53** | 996.0501 | 2.4799 | 1.4496 | 58.5877 |
| **54** | 1009.2700 | 3.0134 | 1.8085 | 7.7088 |
| **55** | 1022.1680 | 3.3227 | 2.0454 | 15.0862 |
| **56** | 1044.4160 | 2.7069 | 1.7397 | 7.0240 |
| **57** | 1061.8900 | 2.1364 | 1.4194 | 0.3610 |
| **58** | 1067.1490 | 1.6378 | 1.0989 | 4.2590 |
| **59** | 1080.2050 | 1.5535 | 1.0680 | 43.6374 |
| **60** | 1087.2950 | 1.7576 | 1.2242 | 3.7757 |
| **61** | 1133.6790 | 3.8501 | 2.9154 | 330.8127 |
| **62** | 1135.4920 | 1.2380 | 0.9405 | 0.6717 |
| **63** | 1137.9450 | 1.3321 | 1.0163 | 52.2656 |
| **64** | 1146.3660 | 2.3258 | 1.8008 | 67.7831 |
| **65** | 1152.4730 | 1.4849 | 1.1620 | 0.3338 |
| **66** | 1162.6880 | 2.3420 | 1.8653 | 12.0823 |
| **67** | 1186.6570 | 1.9314 | 1.6024 | 9.2230 |
| **68** | 1197.2580 | 1.5944 | 1.3465 | 4.7476 |
| **69** | 1198.7450 | 1.3454 | 1.1391 | 905.0415 |
| **70** | 1242.7870 | 1.3610 | 1.2386 | 0.4867 |
| **71** | 1255.1630 | 1.2009 | 1.1147 | 8.6813 |
| **72** | 1267.1390 | 2.8335 | 2.6805 | 51.7245 |
| **73** | 1268.5880 | 1.2666 | 1.2009 | 1.6701 |
| **74** | 1295.4690 | 2.5841 | 2.5551 | 987.2576 |
| **75** | 1299.6740 | 1.2580 | 1.2520 | 1.4721 |
| **76** | 1313.4920 | 1.1577 | 1.1768 | 18.9608 |
| **77** | 1324.7030 | 1.0967 | 1.1339 | 12.3443 |
| **78** | 1333.0200 | 1.2157 | 1.2728 | 36.3769 |
| **79** | 1342.2420 | 1.5131 | 1.6062 | 103.5930 |
| **80** | 1346.5510 | 1.2592 | 1.3453 | 3.2104 |
| **81** | 1373.7960 | 1.4866 | 1.6530 | 0.5913 |
| **82** | 1374.8940 | 5.1324 | 5.7162 | 19.7524 |
| **83** | 1390.4600 | 1.5747 | 1.7937 | 31.8408 |
| **84** | 1395.3410 | 2.8879 | 3.3128 | 403.2864 |

| No | Frequencies [cm$^{-1}$] | Reduced mass [AMU] | Force constants [mDyne/Å] | IR intensity [km/mol] |
|---|---|---|---|---|
| **85** | 1402.0890 | 1.4918 | 1.7279 | 4.8766 |
| **86** | 1410.3020 | 1.2545 | 1.4700 | 3.5627 |
| **87** | 1411.8690 | 1.4154 | 1.6623 | 22.6769 |
| **88** | 1415.3590 | 1.3574 | 1.6021 | 7.0071 |
| **89** | 1450.3570 | 1.2573 | 1.5583 | 4.5610 |
| **90** | 1469.3680 | 2.2861 | 2.9081 | 20.7738 |
| **91** | 1478.0940 | 1.0667 | 1.3731 | 0.1962 |
| **92** | 1483.8320 | 1.0385 | 1.3471 | 0.6324 |
| **93** | 1485.0500 | 1.0724 | 1.3934 | 2.0033 |
| **94** | 1486.9630 | 1.2170 | 1.5854 | 55.9909 |
| **95** | 1488.3190 | 1.0638 | 1.3884 | 0.3907 |
| **96** | 1489.6450 | 1.0460 | 1.3675 | 20.1267 |
| **97** | 1495.9160 | 1.0405 | 1.3718 | 8.9571 |
| **98** | 1498.4840 | 1.0732 | 1.4198 | 10.5941 |
| **99** | 1499.8980 | 1.0814 | 1.4334 | 5.7480 |
| **100** | 1501.9700 | 1.0620 | 1.4116 | 3.4284 |
| **101** | 1507.5870 | 1.0768 | 1.4420 | 19.1450 |
| **102** | 1509.8610 | 1.0766 | 1.4461 | 8.0158 |
| **103** | 1510.4330 | 1.1304 | 1.5195 | 3.3381 |
| **104** | 1526.5790 | 1.1242 | 1.5436 | 79.3379 |
| **105** | 1560.2640 | 2.5834 | 3.7055 | 268.4185 |
| **106** | 1582.7860 | 6.1504 | 9.0782 | 27.2050 |
| **107** | 1639.3640 | 5.5818 | 8.8384 | 1031.4300 |
| **108** | 1689.0450 | 11.2117 | 18.8453 | 588.8097 |
| **109** | 2981.2800 | 1.0837 | 5.6751 | 3.6772 |
| **110** | 3003.0290 | 1.0607 | 5.6360 | 4.1502 |
| **111** | 3003.8060 | 1.0612 | 5.6412 | 2.6860 |
| **112** | 3005.9180 | 1.0400 | 5.5367 | 112.8523 |
| **113** | 3009.9400 | 1.0666 | 5.6932 | 18.9500 |
| **114** | 3011.8760 | 1.0674 | 5.7050 | 144.1304 |
| **115** | 3012.6810 | 1.0436 | 5.5807 | 90.6296 |
| **116** | 3023.6780 | 1.0364 | 5.5826 | 50.4438 |
| **117** | 3029.9520 | 1.0411 | 5.6312 | 40.1115 |
| **118** | 3033.5520 | 1.0894 | 5.9068 | 32.6502 |
| **119** | 3038.4690 | 1.0972 | 5.9685 | 18.8009 |
| **120** | 3041.8270 | 1.0623 | 5.7909 | 30.1728 |
| **121** | 3062.4820 | 1.1037 | 6.0991 | 102.0408 |
| **122** | 3063.7610 | 1.1018 | 6.0936 | 36.9144 |
| **123** | 3064.6450 | 1.1016 | 6.0959 | 48.2166 |
| **124** | 3065.2680 | 1.0959 | 6.0668 | 27.2018 |
| **125** | 3089.7940 | 1.1029 | 6.2035 | 96.2116 |
| **126** | 3094.8410 | 1.1022 | 6.2198 | 35.8778 |
| **127** | 3094.9350 | 1.1024 | 6.2213 | 57.4240 |

| No | Frequencies [$cm^{-1}$] | Reduced mass [AMU] | Force constants [mDyne/Å] | IR intensity [km/mol] |
|---|---|---|---|---|
| **128** | 3095.5280 | 1.1089 | 6.2606 | 62.7141 |
| **129** | 3104.1350 | 1.1017 | 6.2544 | 70.3538 |
| **130** | 3145.1660 | 1.0989 | 6.4046 | 3.0332 |
| **131** | 3156.8310 | 1.0962 | 6.4362 | 23.1319 |
| **132** | 3158.3950 | 1.0925 | 6.4212 | 1.3056 |
| **133** | 3180.4530 | 1.0906 | 6.4995 | 0.1561 |
| **134** | 3203.6130 | 1.0919 | 6.6024 | 2.1513 |
| **135** | 3204.0860 | 1.0933 | 6.6130 | 3.1831 |

## II. Supplementary experimental results

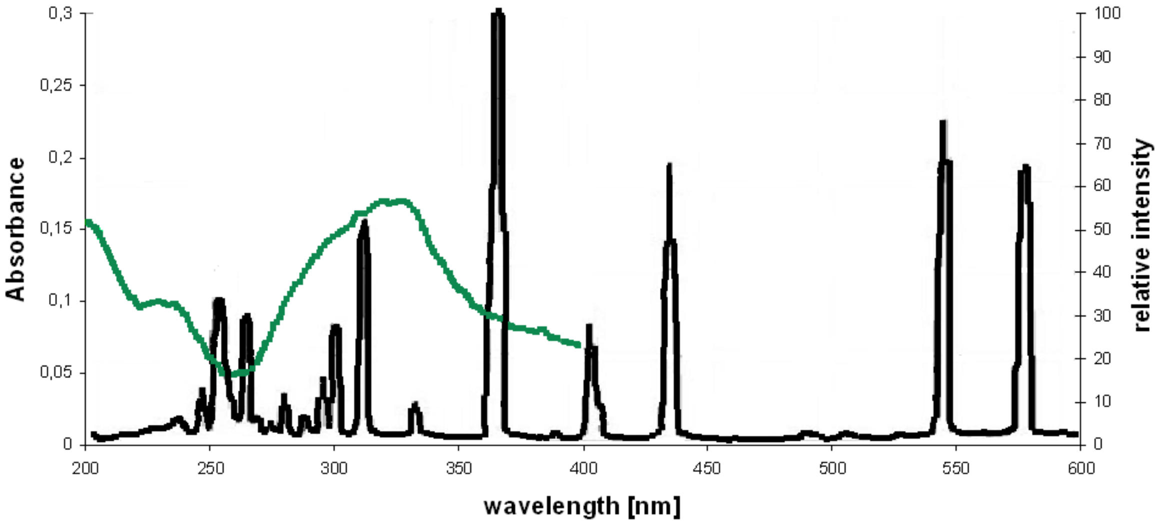


**Fig. S1** UV spectra of ODPABA aqueous solution used in photodegradation experiments (green plot) and provided by the manufacturer emission spectra of Heraeus TQ 150W medium pressure mercury lamp used in the study (black plot)

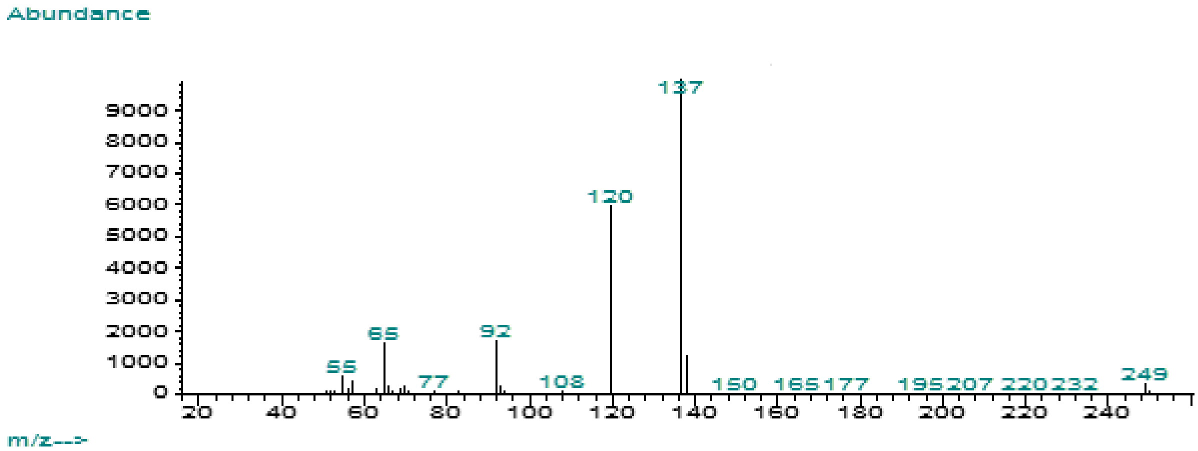


**Fig. S2** Mass spectra of 2-ethylhexyl 4-aminobenzoate identified in ODPABA/UV reaction mixture (retention time, $t_R$=18.02 min.)

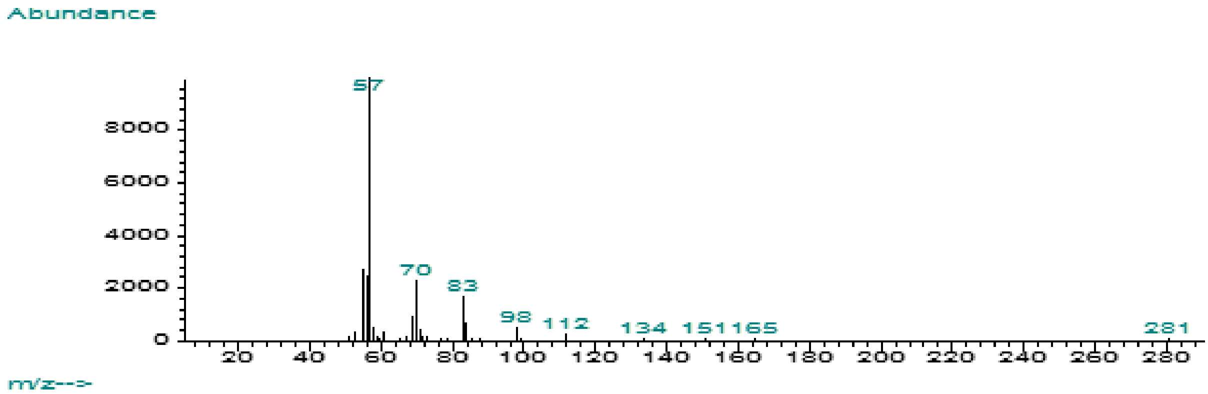


**Fig. S3** Mass spectra of 2-ethyl-1-hexanol identified in ODPABA/UV reaction mixture (retention time, $t_R$=4.17 min.)

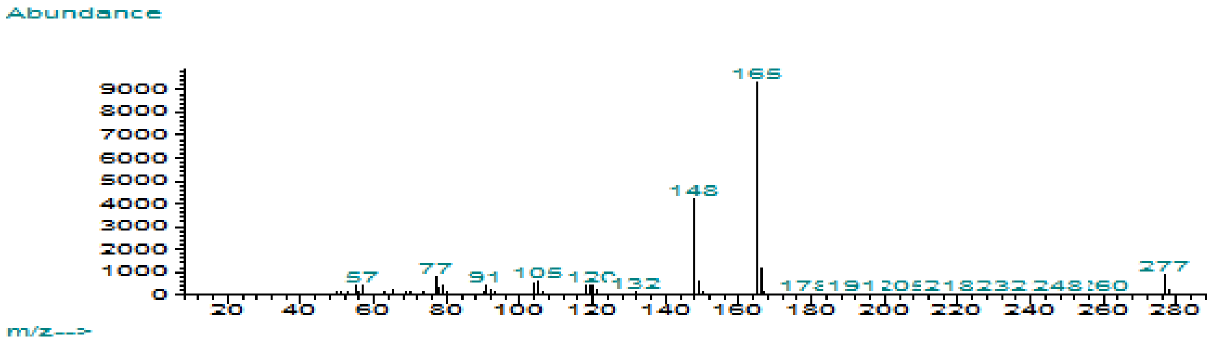


**Fig. S4** Mass spectra of ODPABA identified in ODPABA/$H_2O_2$ reaction mixture (retention time, $t_R$=19.44 min.)

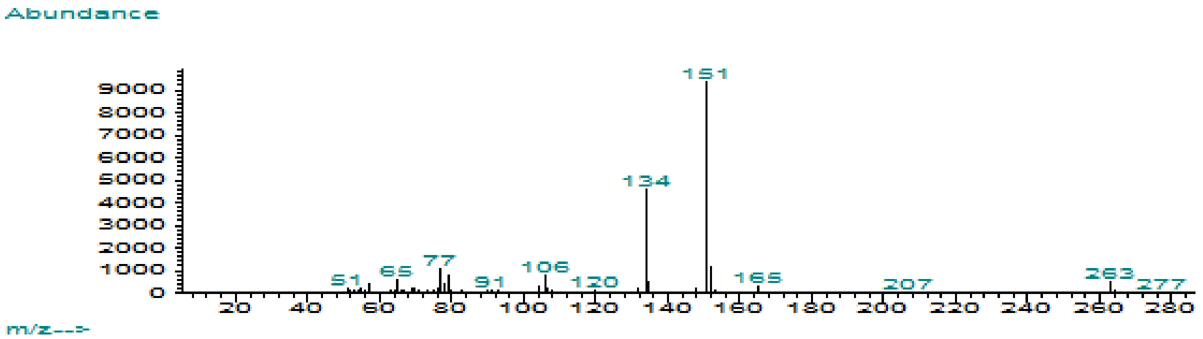


**Fig. S5** Mass spectra of 2-ethylhexyl 4-(methylamino)benzoate identified in ODPABA/$H_2O_2$ reaction mixture (retention time, $t_R$=19.11 min.)

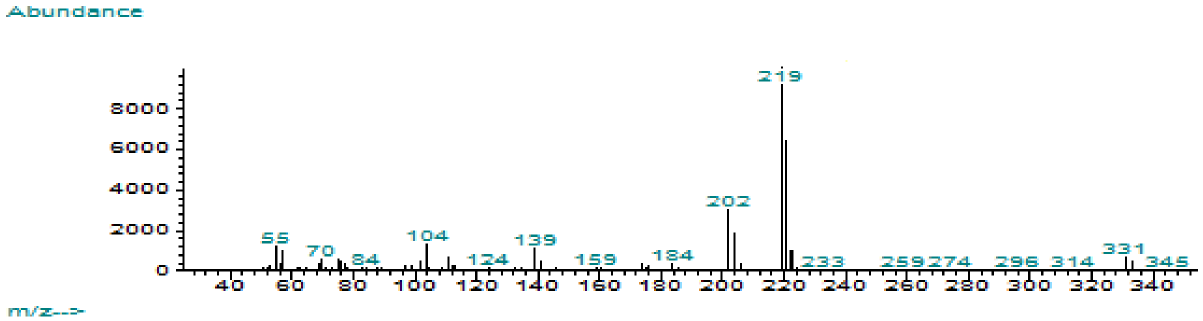


**Fig. S6** Mass spectra of dichlorinated 2-ethylhexyl 4-(methylamino)benzoate identified in ODPABA/NaOCl reaction mixture (retention time, $t_R$=20.38 min.)

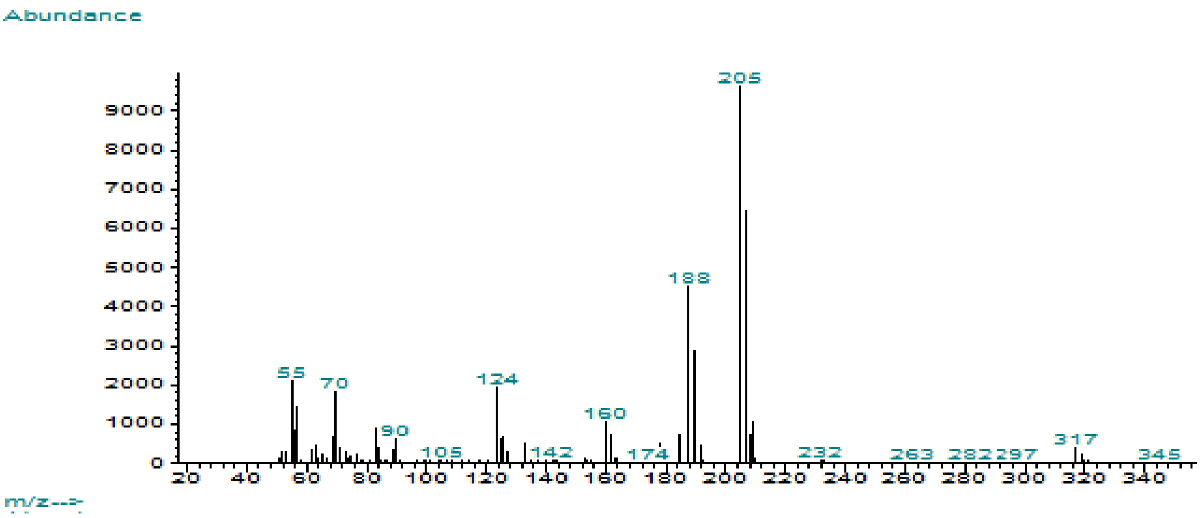


**Fig. S7** Mass spectra of dichlorinated 2-ethylhexyl 4-aminobenzoate identified in ODPABA/NaOCl reaction mixture (retention time, $t_R$=19.79 min.)

## III. Resonance structures

Selected resonance structures of potential ODPABA demethylation transitional product are presented below (Fig. S7).

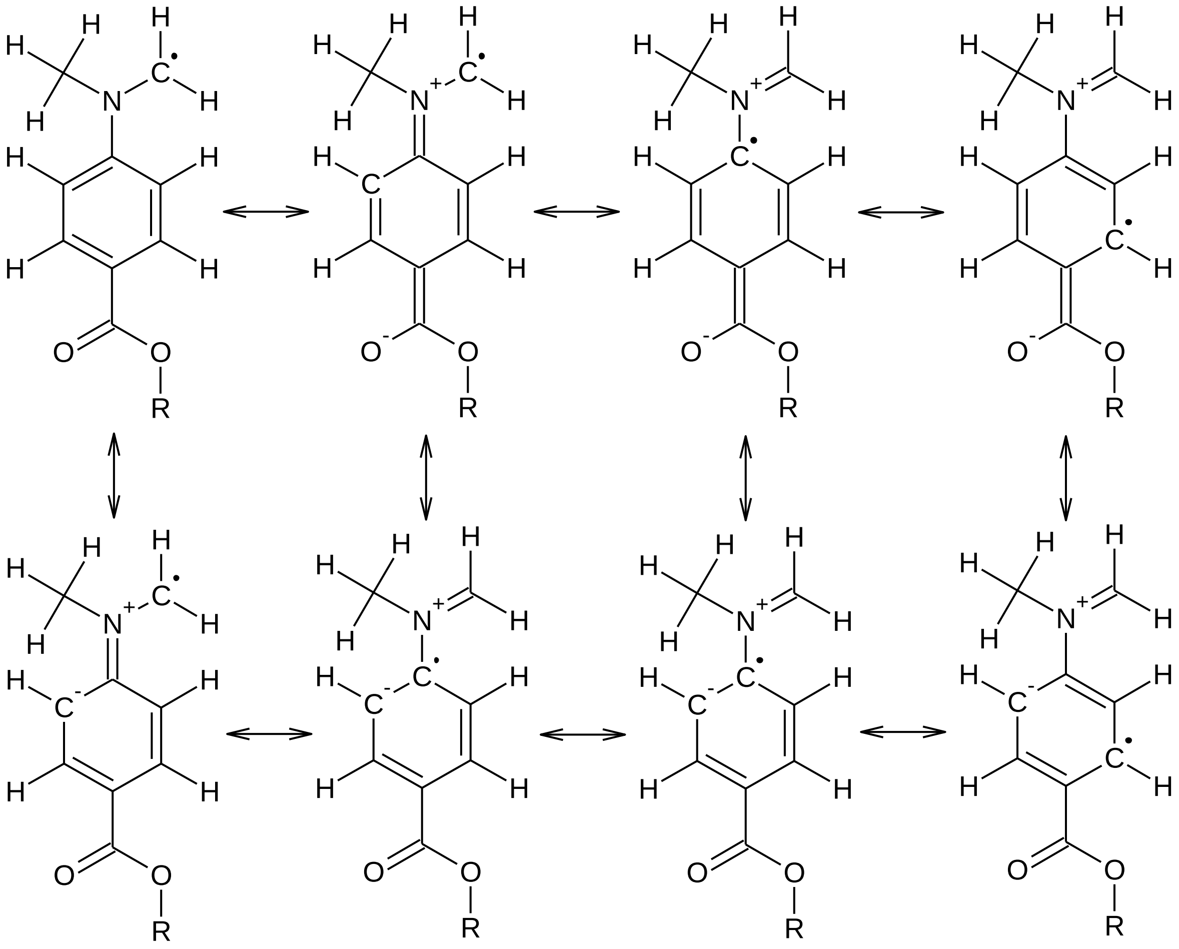

**Fig. S7** The selected resonance structures of the radical formed through hydrogen abstraction from ODPABA ($R=CH_2CH(C_2H_5)C_4H_9$-n)
